# Identification of mosquito larvicidal bacterial strains isolated from north Sinai in Egypt

**DOI:** 10.1186/2191-0855-2-9

**Published:** 2012-01-26

**Authors:** Ferial M Rashad, Waleed D Saleh, M Nasr, Hayam M Fathy

**Affiliations:** 1 Department of Microbiology, Faculty of Agriculture, Cairo University, Giza 12613, Egypt; 2 Department of Microbiology, National Center for Radiation Research and Technology, Nasr city 11371, Egypt

**Keywords:** *Bacillus sphaericus*, PCR, Sequencing, Conventional media, *Culex pipiens*, Larvicidal activity

## Abstract

In the present study, two of the most toxic bacterial strains of *Bacillus sphaericus *against mosquito were identified with the most recent genetic techniques. The PCR product profiles indicated the presence of genes encoding *Bin A, Bin B *and *Mtx1 *in all analyzed strains; they are consistent with protein profiles. The preliminary bioinformatics analysis of the binary toxin genes sequence revealed that the open reading frames had high similarities when matched with nucleotides sequence in the database of other *B. sphaericus *strains. The biological activity of *B. sphaericus *strains varied according to growing medium, and cultivation time. The highest yield of viable counts, spores and larvicidal protein were attained after 5 days. Poly (P) medium achieved the highest yield of growth, sporulation, protein and larvicidal activity for all tested strains compared to the other tested media. The larvicidal protein produced by local strains (*B. sphaericus *EMCC 1931 and EMCC 1932) in P medium was more lethal against the 3^rd ^instar larvae of *Culex pipiens *than that of reference strains (*B. sphaericus *1593 and *B. sphaericus *2297). The obtained results revealed that P medium was the most effective medium and will be used in future work in order to optimize large scale production of biocide by the locally isolated *Bacillus sphaericus *strains.

## Introduction

Mosquito borne diseases constitute a serious health hazard to human. It has been established that, mosquito's females as blood sucking insects, are vectors of a multitude disease of man and animals in different countries through transmission of pathogenic agents. Mosquitoes are belonging to the order *Diptera *and family *Culicidae *which include the genera of medical importance, *Aedes, Anopheles, Culex *and *Mansonia*. At least 90% of the world malaria (*Anopheles*), yellow fever (*Aedes*), dengue (*Aedes*), encephalitides (*Aedes*) and lymphatic filariasis (*Aedes, Anopheles *and *Culex*) occurs in the tropics where the environmental conditions favor insect vectors responsible for the transmission of diseases Rawlins, 1989.

Controlling insect populations with chemical insecticides has proven useful. Over time, mosquitoes developed resistance to chemical insecticides, toxicity to non target organisms, increased public awareness of the toxicity hazards, undermined this control strategy's efficacy. Within this scenario, biological control based on insecticidal bacteria has proven effective in controlling insect vectors. Mosquitocidal *Bacillus thuringiensis *subsp. *israelensis *and *Bacillus sphaericus *are used as an alternative for synthetic chemical insecticide in controlling larvae of mosquitoes over two decades. *B. thuringiensis *subsp. *israelensis *has a wider spectrum of activities against *Anopheles, Culex *and *Aedes *spp; while the target spectrum of *B. sphaericus *is restricted mainly to *Culex*, for a lesser extent to *Anopheles *and only few *Aedes *species. Compared to *B. thuringiensis *subsp. *israelensis*, the popular microbial mosquito control agent, *B. sphaericus *has major advantage. It appears to persist in the environment longer especially in polluted water, and thus can establish a longer lasting control of larval populations.

The toxicity of *B. sphaericus *strains is mainly attributed to the presence of binary toxin (*Bin A, Bin B*) and/or mosquitocidal (*Mtx*) toxin genes. Binary toxin is comprised of two polypeptides of 42- and 51-kDa and produced during sporulation. The other group of toxins (mtx1, mtx2, mtx3) is produced during vegetative growth. Highly toxic strains of *B. sphaericus *contains btx as principle factor or both btx and mtx, whereas the weakly toxic strains only contain mtx genes Charles *et al.*, 1996.

Despite the excellent performance of *B. sphaericus *in the field, the presence of only the Bin toxin in spores as the major toxic moiety of commercial preparation has allowed insects to develop resistance Yuan *et al.*, 2000 that may limit its application or necessitate rotation with other insecticides.

A program on biological control of mosquitoes, virulence prospecting and evaluation of new isolates around the world is one of the most important steps taken to determine their effect on target populations and thereby selecting the most promising strains for producing biological insecticides Litaiff *et al.*, 2008. Since the use of locally available effective strains are always advisable in insect control programs, the search for more effective strains able to overcome this resistance should be continued with emphasis on the isolation of more toxic strains. In an earlier study, Fathy (2002) isolated and morphologically and biochemically characterized a number of highly toxic bacterial strains against mosquito. The aim of the present investigation is to further identify two potent of the isolated strains using modern genetic techniques. Selection of the best medium that markedly supports active cell growth and high biocide production yield will also be considered.

## Materials and methods

### Microorganisms

Two actively marked toxic strains of *Bacillus sphaericus *(Fathy, 2002), previously isolated from the soil of north Sinai in Egypt, identified morphologically, biochemically and assayed biologically against mosquito larvae (strains are available in Culture collection of Cairo "MIRCIN" under numbers EMCC 1931 and EMCC 1932, Agric. Faculty, Ain Shams University). They were used in the present study along with the reference strains of *B. sphaericus *1593 and 2297 as highly toxic strains Charles *et al.*, 1996. The reference strains, *B. sphaericus*1593 and *B. sphaericus *2297, were kindly provided by Prof. Dr. Y. A. Osman, Mansoura University and Prof. Dr. M. S. Foda, National Research Center, respectively.

#### Maintenance of microorganisms

Stock cultures were maintained in heavy spore suspensions at 4°C until required.

### Characterization of the selected local strains

#### Polymerase chain reaction and primer sequences

##### Purification of genomic DNA

Total DNA was prepared from bacterial strains according to the methodology of Sambrook *et al. *(1989). Each *B. sphaericus *strain was grown overnight in a 100 ml of Luria Bertani LB medium Bertani, 1951 at 30°C. Cells were harvested by centrifugation at 6000 rpm and 4°C for 10 min and washed with distilled water. The pellets were frozen at - 80°C for 1 h then thawed at 37°C; resuspended in 5 ml of solution containing 2 mg/ml lysozyme and incubated for 1 h at 37°C. Then, 0.5 ml of sodium dodecyl sulphate, SDS, (1%) was added and the solution was left for 15 min at room temperature. The cell lysate was mixed with equal volume of phenol/chloroform and kept on ice for 5 min followed by spinning for 20 min at 10000 rpm and 4°C. The supernatant that contains the DNA is mixed again with equal volume of phenol/chloroform for 5 min on ice to get rid of any remaining proteins and respinning for 20 min at 10000 rpm and 4°C. Afterward, 0.1 volume of sodium acetate (3 M) and 2.5 volume of absolute ethanol was added. DNA was collected by centrifugation and the pellet was dried and washed with 70% ethanol. The DNA pellet was collected again, dried and 50 μl TE buffer was added and mixed well. Finally, 10 μl of RNase was added to the DNA solution and left at 37°C for 2 days in order to remove any contaminating RNA.

##### Primer

According to the published sequences of the *B. sphaericus *toxin genes Shanmugavelu *et al.*, 1995, sequences for three sets of primers of the toxin genes were selected; synthesized and obtained from **Biobasic, Canada **and then used for PCR amplification. The sequences of three pairs of specific primers were used to identify genes *Bin A, Bin B *that encode binary toxins (41.9 and 51.4 kDa) and gene *Mtx1 *that encode mosquitocidal toxin, 100 kDa; the expected size of amplified products are shown in Table 1.

**Table 1:** The sequence of primers and the expected size of the amplified products.

Primer	Sequence	Length meres	Standard	Product size
***BinA***	5'ATGAGAAATTTGGATTTTATT 3'	21	1593 M	1.1 kb
		
	5'TTAGTTTTGATCATCTGTAAT 3'	21	1593 M	

***BinB***	5'ATGTGCGATTCAAAAGACAAT3'	21	1593 M	1.3 kb
		
	5'TCACTGGTTAATTTTAGGTA 3'	20	1593 M	

***Mtx1***	5'ATGGCTATAAAAAAAGTATTA3'	21	1593 M	2.6 kb
		
	5'TACTATCTAGGTTCTACACC 3'	20	1593 M	

The suspensions of genomic DNAs were transferred to 25 μl of PCR-reaction mixture containing 0.5 μM of each primer, 0.2 mM of each dNTP, 1× of *Taq *polymerase buffer, 1.5 mM MgCl_2 _and 2.5 U of *Taq *polymerase (Red Hot). The PCR amplifications were performed as follows: initial denaturation of DNA at 94°C for 5 min, 35 cycles comprised of 1 min denaturation at 94°C, 1 min annealing at 55°C, 2 min elongation step at 72°C followed by a final extension step at 72°C for seven min. Amplicons were visualized by electrophoresis on 1% agarose gel stained with ethidium bromide. The banding was visualized at short UV light Carozzi *et al.*, 1991.

##### Sequence analysis

The dideoxyribonucleoside chain termination procedure originally developed by Sanger *et al. *(1977) was employed for sequencing the double-stranded DNA obtained during the PCR. Sequencing was conducted under BigDyeTM terminator cycling condition. The reacted products were purified using Ethanol Precipitation and run using Automatic Sequencer 3730 × l (Macrogen, DNA sequencing, USA). The nucleotide sequence data of the *Bin *toxins open reading frame was submitted to the BLASTN programs search nucleotide data bases http://www.ncbi.nlm.nih.gov.

Sequencing the DNA alignments encoding binary toxins were performed according to EXPASY Proteomics Server (Expert Protein Analysis System) proteomics server of the Swiss Institute of Bioinformatics (SIB). http://www.expasy.org. The partial DNA sequences for Bin A and B from the local strains (*Bacillus sphaericus *EMCC 1931 and 1932) were assigned GenBank accession nos. JN007909, JN0079010 and JN0079011, JN0079012 for *B. sphaericus *EMCC 1931 and *B. sphaericus *EMCC 1932, respectively.

#### Protein profile analysis

The most commonly method of analysis and separation of protein is sodium dodecyle sulfate-polyacrylamide gel electrophoresis (SDS-PAGE) which based on the relative molecular weight of protein Laemmli, 1970. For each strain of *B. sphaericus*, protein analysis was made for both 18 and 120 h cultures. Samples of whole cultures were centrifuged at 6000 rpm and 4°C for 10 min; then the pellets were harvested and washed three times using distilled water (6,000 rpm/10 min/4°C). The pellets resuspended in an equal volume of loaded buffer followed by scratching and heating at 100°C for 5 min., the extracts were clarified by centrifugation at 10,000 rpm for 10 min; the supernatants were injected in polyacrylamide gel for protein separation.

The separating gel solution was prepared and poured into the gel apparatus between the cleaned glass plates. The gel solution was immediately overlaid with water and allowed to polymerize at room temperature for 45-60 min, then water was removed. Stacking gel solution was prepared, poured over the separating gel and allowed to polymerize for 30-45 min. Polyacrylamide gels were stained in Coomassie brilliant blue solution with gentle shaking for up to 3 h at room temperature.

Separation was done using Mini-Protein II electrophoresis unit at 200 V, constant voltage for approximately 45 min in SDS-electrophoresis buffer. The gel was destained by incubation in several volumes of Coomassie brilliant blue destain solution for up to 8 h at room temperature. Proteins were detected as blue-stained bands against clear background.

Protein profile analysis was carried out using Gel Documentation System (Alpha Image 2000), Germany.

#### Cell growth and toxin production by bacterial strains in various cultivation media

*B. sphaericus *strains were grown on nutrient agar slants at 30°C for 72 hr. Seed cultures were carried out following the technique of Obeta and Okafor (1983). The slant cultures were washed with 5.0 ml sterile distilled water, which were then added to 250 ml flasks containing 50 ml nutrient broth. The flasks were placed on a rotary shaker at 200 rpm and incubated for 24 hr at 30°C. From these first- passage seed cultures, 5.0 ml were used to inoculate similar seed flasks and treated as above for 18 h.

Five conventional laboratory media that have been recommended as reference media by many authors were used for *B. sphaericus *production as follow: Glucose-Glutamate-Salts- EDTA (GGSE medium), Chan *et al. *(1972), g/l, glucose 5, monosodium glutamate 10, K_2_HPO_4 _0.5, KH_2_PO_4 _0.5, MgSO_4_.7H_2_O 0.2, FeSO_4_.7H_2_O 0.01, MnSO_4_.4H_2_O 0.01, ZnSO_4_.7H_2_O 0.013, CaCl_2 _0.025, thiamine 0.0005, biotin 1 μg, EDTA 25 μg/ml; Nutrient Yeast Extract Salt (NYS medium) without glucose, Yousten and Davidson (1982), g/l, peptone 5, beef extract 3, yeast extract 0.5, MnCl_2 _0.01, CaCl_2 _0.1, MgCl_2 _0.2; Poly (P medium), Bourgouin *et al. *(1984), g/l, peptone 5, beef extract 5, yeast extract 10, glycerol 10, NaCl, 3; Acetate Yeast Extract (AYE medium), Sasaki *et al. *(1998), g/l, sodium acetate 5.45, yeast extract 10, MnCl_2_. 4H_2_O 0.02, CaCl_2_. 2H_2_O 0.2, MgCl_2_. 6H_2_O, 1.02, KH_2_PO_4 _0.5 and Luria Bertani (LB medium), Poopathi *et al. *(2002) g/l, peptone 5, yeast extract 2.5, NaCl 5. The pH of all media was adjusted to 7.1 ± 0.1 with 1N NaOH, and the media were dispensed in flasks as 20% v/v and sterilized at 121°C for 20 min.

Production flasks of each medium were inoculated in triplicate with 1.0 ml (2% v/v, Prabakaran *et al.*, 2007) of a second passage seed culture of each *B. sphaericus *strains and allowed to grow at 30°C for 5 days on a rotary shaker (Cole Parmer, 51604) at 200 rpm. Culture samples were drawn from each culture medium at 0, 1, 3 and 5 days intervals.

##### Total viable and spore counts

Serial decimal dilutions of culture samples were prepared; 1 ml of each dilution (in triplicates) was added to Petri dish, followed by addition of nutrient agar medium. For spore counts, the serial dilutions of culture samples were pasteurized at 80°C for 15 min before plating. Plates were incubated at 30°C for 48 h and the developing *B. sphaericus *colonies were counted and expressed as cfu/ml and/or spores/ml. The pH of culture samples were estimated using a digital pH meter (JEN WAY, 3305).

##### Biochemical studies and toxicity bioassay

Whole culture samples for each strain on different media were centrifuged at 6000 rpm and 4°C for 10 min and washed twice with distilled water. The pellets resuspended in distilled water and used for protein determination and toxicity bioassay.

##### Protein determination

Protein extracts were prepared by adding 25 μl of 2 M NaOH solution to each ml suspension followed by incubation at 37°C for 3 hr Sasaki *et al.*, 1998. After centrifugation and extraction as mentioned above, protein concentrations in the clarified supernatant were determined using the technique of Bradford (1976) with bovine serum albumin (BSA, Sigma) as standard.

#### Bioassay against *Culex pipiens *larvae

The *Culex pipiens *3^rd ^instar larvae were obtained from mosquito rearing laboratory in Research Institute of Medical Entomology, Ministry of Health. Serial dilutions of the previously resuspended pellets were prepared in distilled water, and then one ml of each dilution was added to 100 ml distilled water in 200 ml plastic cups. Twenty, 3^rd ^instar larvae of *C. pipiens *were placed in each cup and suitable amount of larval food was added (ground dried bread: dried Brewer's yeast as 2:1). Experiments were conducted at room temperature of 28°C ± 2. Each experiment included 3 concentrations in triplicates, as well as appropriate control. Larval mortality was scored after 48 h and corrected (if needed) for control mortality using Abbott's formula Abbott, 1925.

### Statistical analysis

All data were statistically analyzed using Factorial ANOVA test MSTAT-C Version 4, 1987

## Results

### Characterization of the locally isolated strains

#### Detection of toxin genes by Polymerase chain reaction (PCR)

**Figure 1:**
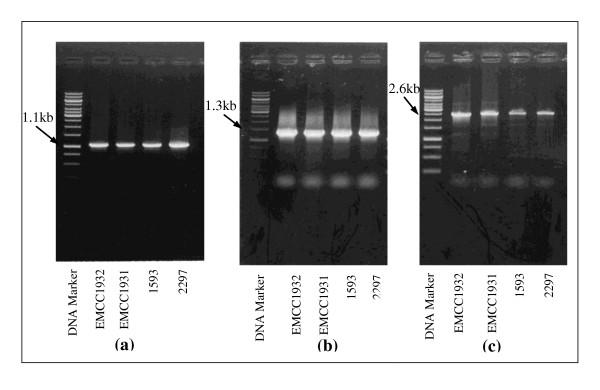
**PCR product profiles of *Bacillus sphaericus *strains**.

The expected sizes of the PCR products were 1.1, 1.3 and 2.6 kb for *Bin A, Bin B *and *Mtx1 *toxin genes, respectively. As shown in Figure (1a, b and 1c), the primer designed for each gene amplified the target toxin gene as the amplicon obtained was of the expected size. The PCR product profiles of the local strains (*Bacillus sphaericus *EMCC 1931 and EMCC 1932) are identical to those of the reference strains, *B. sphaericus*1593 and *B. sphaericus *2297. Analysis of these profiles proved that both the standard and local strains harbor the *Bin A, Bin B *and *Mtx1 *genes encoding Bin A 42-, Bin B 51- and Mtx1 100-kDa proteins.

#### Sequencing of the binary toxin gene operons from local strains

**Figure 2:**
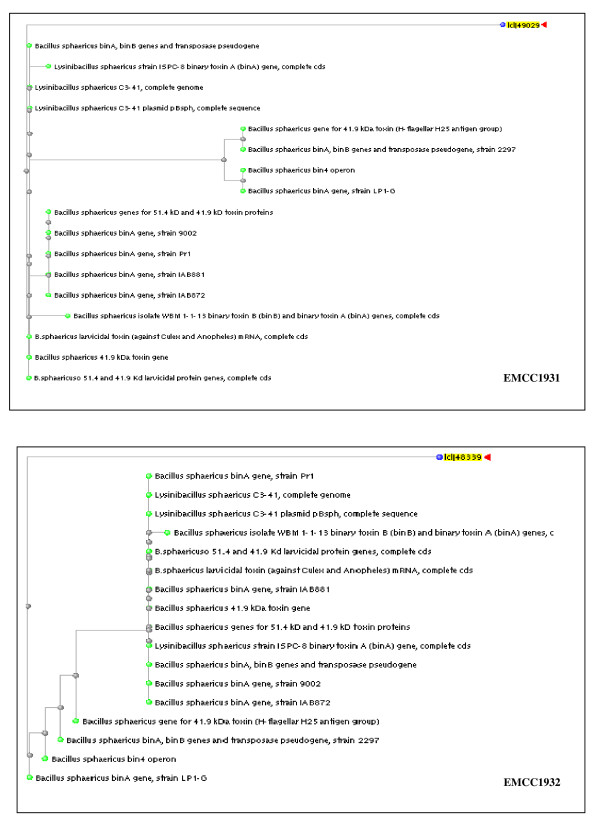
**Phylogenetic trees of the DNA fragments of *Bin A *genes encoding 42-kDa toxin of local strains of *B. sphaericus***.

**Figure 3:**
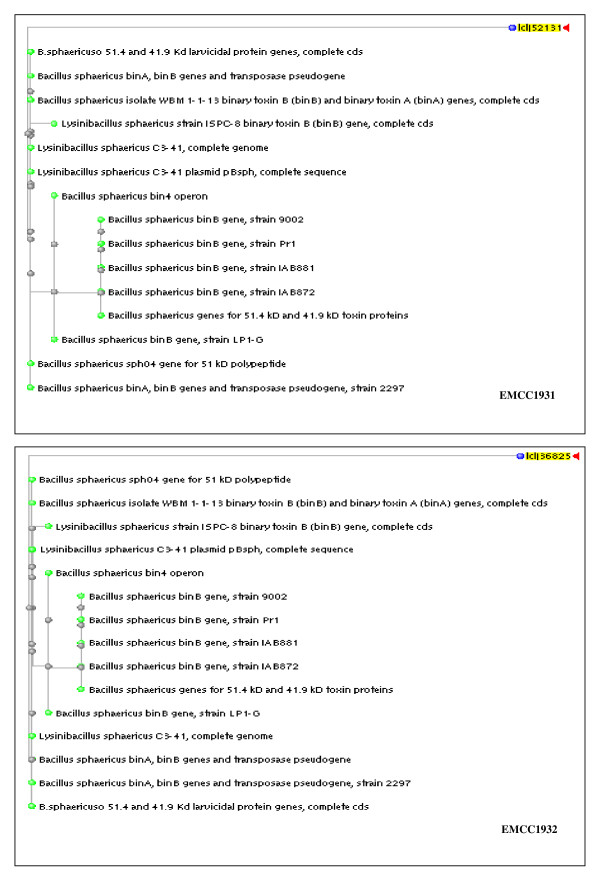
**Phylogenetic trees of the DNA fragments of *Bin B *genes encoding 51-kDa toxin of local strains of *B. sphaericus***.

The preliminary bioinformatics analysis of the binary toxin genes sequence revealed that the open reading frames had high similarities when matched with nucleotides sequence in the database of other *B. sphaericus *strains. Based on linear DNA sequences of Bin A (737 bp and 348 bp) and Bin B (513 bp and 373 bp) of local strains (*B. sphaericus *EMCC 1931 and EMCC 1932) respectively, the phylogenetic trees (Figure 2 &3) were constructed. Genetically, *Bin A *and *B gene *operons from the local strain *B. sphaericus *EMCC 1931 were found to be close to those from other *B. sphaericus *strains with at least 94% similarities. However, the sequences of binary toxin gene operons from the other local strain *B. sphaericus *EMCC 1932 revealed lower similarity (only 81% for *Bin A *and 89% for *Bin B *genes). The locus sequences of DNA linear Bin A and Bin B binary toxin genes, partial cds were deposited in the GenBank under the accession numbers JN007909, JN0079010, and JN0079011, JN0079012 for *B. sphaericus *EMCC 1931 and EMCC 1932, respectively.

**Figure 4:**
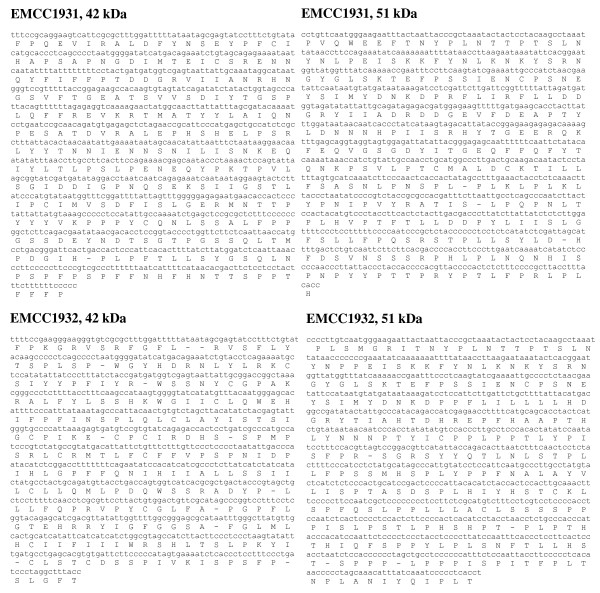
**Nucleotide sequence of the DNA fragment encoding 42 kDa and 51 kDa toxins of local strains of *B. sphaericus *EMCC1931 and EMCC1932**. The predicted amino acid sequence is given in the single-litter code.

**Figure 5:**
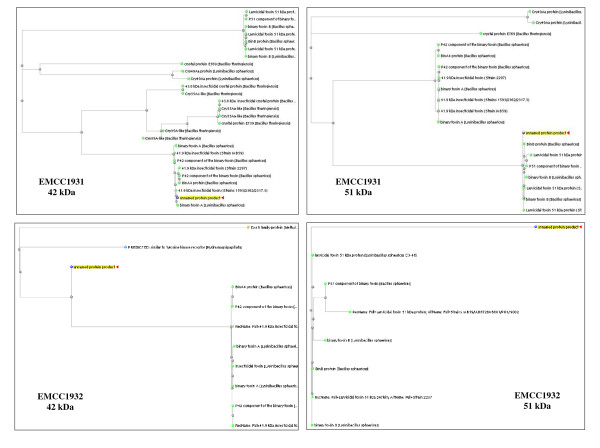
**Similarity trees of the DNA fragments of *Bin A *and *Bin *B genes encoding 42- kDa and 52-kDa toxins of local strains of *B. sphaericus *EMCC1931 and EMCC1932**.

Derived from the nucleotide sequence of the DNA fragments encoding the 42- and 51-kDa toxin proteins of the local strains (*B. sphaericus *EMCC 1931 and EMCC 1932), the amino acid sequences were deduced and shown below the nucleotide sequence; the constructed similarity trees were drawn (Figure 4 &5). It is apparent that the 42- and 51-kDa toxin proteins in local strains have high levels of similarity when matched with protein in binary toxins in the database. A significant homology of 95% was found between the amino acid sequence of the *B. sphaericus *EMCC 1931 protein and the 42- kDa toxin over a stretch of 283 amino acids. This percentage of identity was markedly decreased to 81% in case of the protein produced from *B. sphaericus *EMCC 1932 strain over 254 amino acids. With respect to 51-kDa toxin protein, the amino acid sequence obtained from the *B. sphaericus *EMCC 1931 protein recorded a similarity percentage of 94, overall 279 amino acids. On the other hand, this similarity decreased to record 89% with the second strain, *B. sphaericus *EMCC 1932, with 268 amino acids.

#### Analysis of protein profiles by SDS-PAGE

**Figure 6:**
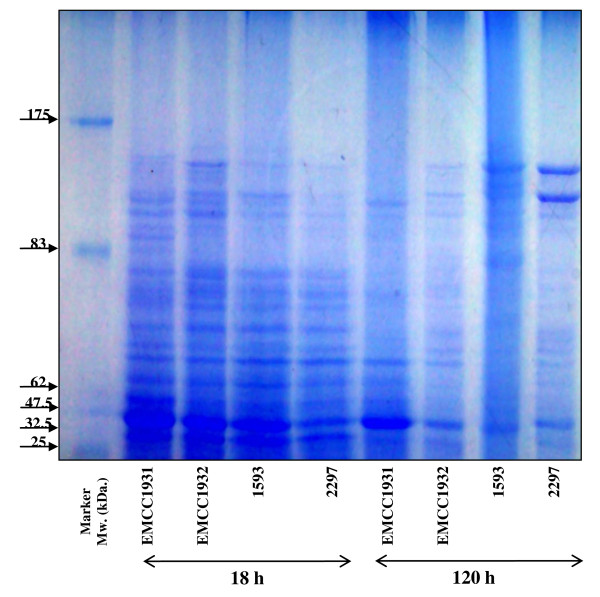
**Gel electrophoresis of crude toxin extracts from *B. sphaericus *strains**. Molecular weight standards in kDa: 175, 83, 62, 47.5, 32.5 and 25.

**Figure 7:**
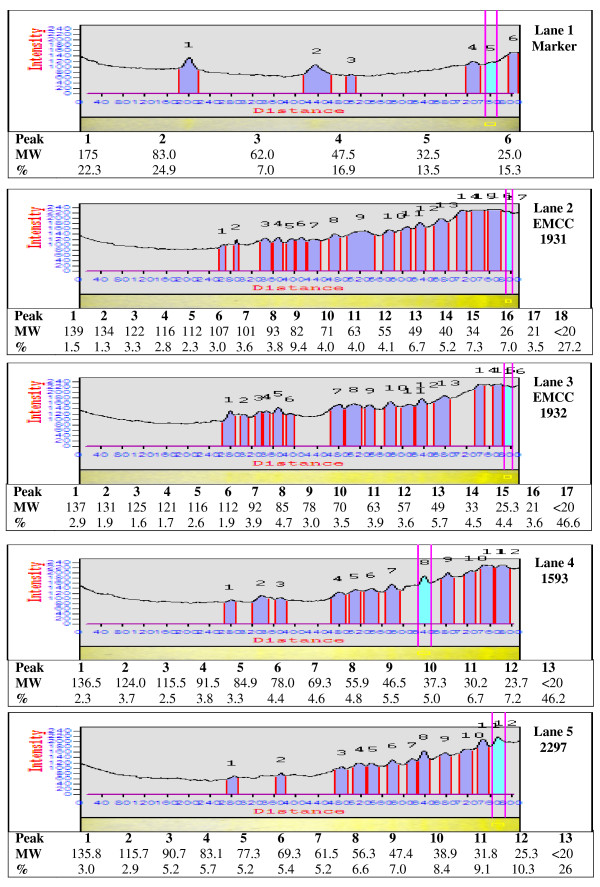
**Electrophoretic pattern of protein fraction in the local and reference strains of *Bacillus sphaericus *(18 h old)**.

**Figure 8:**
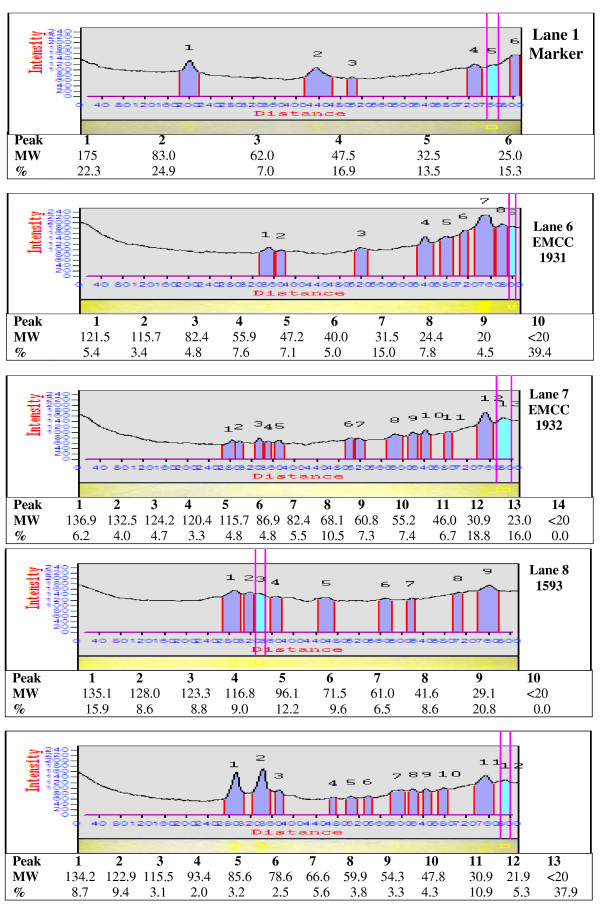
**Electrophoretic pattern of protein fraction in the local and reference strains of *Bacillus sphaericus *(120 h old)**.

The protein patterns of the local (*B. sphaericus *EMCC 1931 and EMCC 1932) and reference (*B. sphaericus *1593 and *B. sphaericus *2297) strains resulting from SDS-PAGE (Figure 6) showed that numerous molecular weights of proteins were detected for both 18 and 120 h cultivation. The protein fractions separated along 9 - > 17 bands with molecular weights ranged from 20- to 139- kDa were quantitatively differed as estimated from their migration in SDS-PAGE, densitographs (Figures 7 &8). The high molecular mass protein bands over the range of 128- to 139- kDa were detected in all lanes from 18 and 120 h cultures, however, disappeared after 120 h only in the culture of *B. sphaericus *EMCC 1931. Also, bands of protein fractions with 110- to 125-kDa were found in the lanes of all cultures during the vegetative growth and after the completion of sporulation. Only two bands of protein fractions with molecular weight 100- to 107-kDa were found in the lane of 18 h culture of the strain *B. sphaericus *EMCC 1931. An additional bands at about 90 - 94-, 82 - 85-, 67- 78-, 54 - 63-, 40 - 48-, 30 - 38- < 20 -29 kDa were observed in all lanes with minor exceptions between 18 and 120 h.

#### Cell growth and toxin production by bacterial strains in various cultivation media

**Figure 9:**
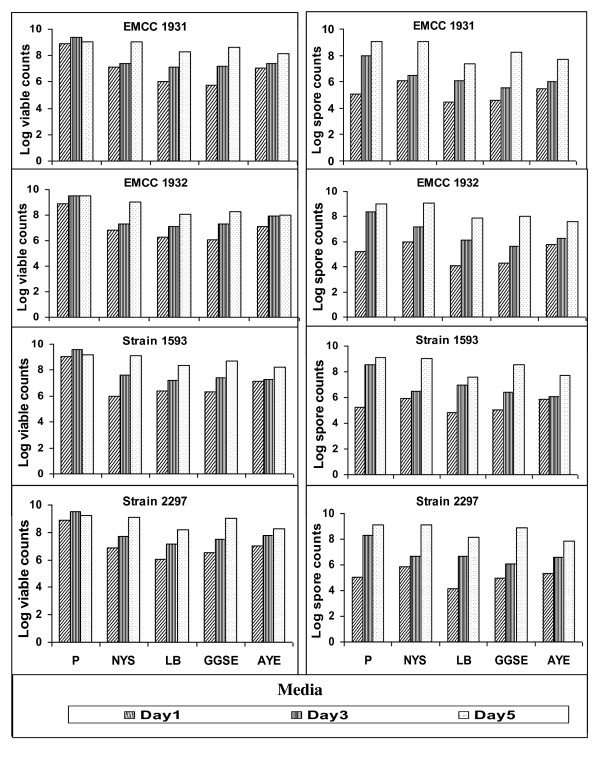
**Total viable (LSD_0.01 _= 2.15, CV = 1.43%) and spore (LSD_0.01 _= 1.57, CV = 1.27%) counts of local (EMCC 1931, EMCC1932) and reference (1593, 2297) strains of *B. sphaericus *in different conventional laboratory media during cultivation course**.

Regarding, the overall growth and toxin production in the tested laboratory media, it is palpable that the viable counts of *B. sphaericus *strains varied according to growing medium, P medium had the highest viable counts for all strains throughout the cultivation time (Figure 9). After 24 h cultivation, P medium achieved the highest significant counts (8.7 × 10^8 ^- 1.2 × 10^9 ^cfu/ml), however no significant differences could be observed between the other tested media. Increasing cultivation time up to 72 h resulted in increasing growth yield; P medium was still the best medium supporting the growth of different *B. sphaericus *strains (about 43 - 300 fold over the other tested media). After 5 days and at the end of the cultivation course, the viable counts of different strains, to some extent, remained stable in P medium, but they increased in the other tested media with multiplication rates ranged from 1.25 to 58.3 fold. NYS medium attained the highest significant yield of 1.1 - 1.3 × 10^9 ^cfu/ml and stand with P medium without significant differences. GGSE medium (2.0 × 10^8 ^- 1.0 × 10^9 ^cfu/ml), LB medium (1.2 - 2.7 × 10^8 ^cfu/ml) and AYE medium (1.0 - 1.8 × 10^8 ^cfu/ml) came after (Figure 9).

Sporulation rate was generally at low levels after 24 h in all tested media, P medium recorded the lowest significant rate (1.3 - 1.7%), although it attained the highest viable spore counts. After 72 h, sporulation rate was still low (Figure 9) and significant differences were observed within the tested strains in P medium and between P medium and the other tested media. Consequently, the maximum yield of spores was reached at the end of the cultivation time in both P medium and NYS medium (1.0 - 1.4 × 10^9 ^spores/ml) followed by GGSE medium (1.1 - 8.0 × 10^8 ^spores/ml), however, the highest significant sporulation rate (88.5 - 100%) was obtained in NYS medium followed by P medium and GGSE medium (71.4 - 93.3%), (40.0 - 80%) in that order. There were no significant differences between the local (*B. sphaericus *EMCC1931) and the reference (*B. sphaericus *2297) strains but they varied significantly with the other tested strains (Figure 9).

**Figure 10:**
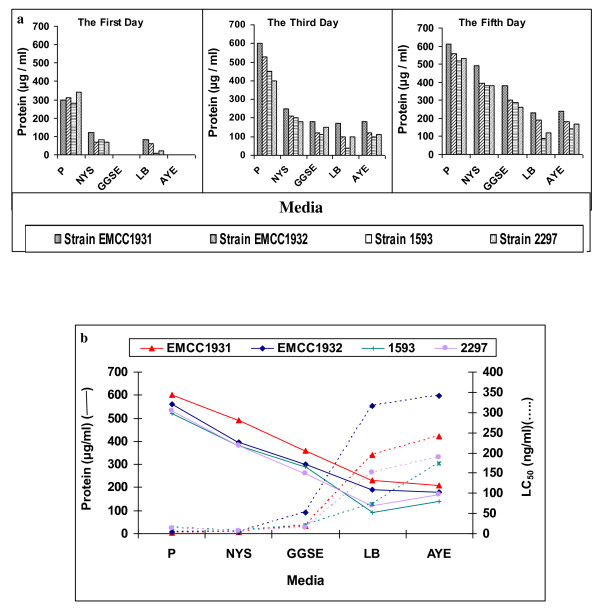
**a) Protein synthesis (LSD _0.01 _= 0.09, CV = 0.02%) in the conventional media during the incubation time, b) Relation between protein synthesis (LSD_0.01 _= 2.21, CV = 0.32%) and mosquitocidal activities (LSD_0.01 _= 1.37, CV = 0.68%) of local (EMCC1931, EMCC1932) and reference (1593, 2297) strains of *B. sphaericus *at the end of incubation time**.

Protein was produced after 24 h in the cultivation media except in GGSE medium and AYE medium, whereas it was produced lately. Protein was in increasing order along the cultivation course. The highest significant quantities were attained in P medium by the local strains (*B. sphaericus *EMCC 1931 and EMCC 1932) by 5 days (Figure 10a). Generally, the quantities of produced protein differed significantly among the tested media and strains along the cultivation time. Local strain, EMCC 1931 always produced significantly higher protein amounts in cultivation media than standard strains 2297 and 1593.

The larvicidal activities of the tested strains were performed with toxins produced from different laboratory media; the lethal concentrations were expressed as nanogram of produced protein/ml. The comparative toxicities of *B. sphaericus *strains produced from five culture media are shown in Figure 10b. It is clearly observed that protein produced by local strains (*Bacillus sphaericus *EMCC 1931 and EMCC 1932) in P and NYS media was more lethal than that of reference strains (*B. sphaericus*1593 and *B. sphaericus *2297). The lowest LC_50 _values of 2.5, 3.2, 12 and 13.8 ng/ml were achieved in poly medium after the 5^th ^day by *B. sphaericus *strains EMCC 1931, EMCC 1932, 1593 and 2297, respectively, against the 3^rd ^instar larvae of *Culex pipiens *(Figure 10b). However, the LC_50 _values of 4.4, 4.8, 6.1 and 7.2 ng/ml were obtained in NYS medium, in that order.

Significant differences in toxicity against mosquito larvae were observed between P and NYS media, local (*Bacillus sphaericus *EMCC 1931, EMCC 1932) and reference (*B. sphaericus*1593, *B. sphaericus *2297) strains. With the rest of media, significant differences were observed between all tested strains and media. The lowest toxicity was observed in LB and AYE media, respectively.

pH values was in increasing order in all cultivation media as a result of the growth of different strains. The values of final pH in all tested cultures ranged between 8.4 and 9.0.

## Discussion

Since the use of locally available effective bacterial strains is always advisable in insect control programs, it should be genetically analyzed to screen the presence of newer or perhaps more toxic stains. The PCR analysis of new isolates provides a valuable prescreen that permits their prioritization for subsequent insect assays.

In the current study, PCR analysis proved that both the standard 1593, 2297 and local EMCC 1931, EMCC1932 strains of *B. sphaericus *harbor the *BinA, BinB *and *Mtx1 *genes encoding Bin A 42-, Bin B 51- and Mtx1 100-KDa proteins. This result is in accordance with the information provided for *BinA, BinB *and *Mtx1 *toxin genes Shanmugavelu *et al.*, 1995; Charles *et al.*, 1996; Otsuki *et al.*, 1997; and the description of *B. Sphaericus *2297 and 1593 as highly toxic strains. Binary toxin genes are considered major factors for mosquito larvicidal activity. More recently, Jagtap *et al. *(2009) proved that the highly toxic *B. Sphaericus *2297 and 1593 strains have five toxin genes encoding mosquito larvicidal toxins, namely *BinA, BinB, Mtx1, Mtx2 *and *Mtx3*.

The obtained bioassay results confirmed the superiority of the locally isolated strains compared to the two reference strains with respect to toxicity against mosquito larvae. To assess whether this toxic activity is attributed to a new variant of the bin operon, the amplified PCR products of bin genes were sequenced. Based on bioinformatics analysis, the overall nucleotide and protein similarities veritably indicated that both *B. sphaericus *EMCC 1931 and EMCC 1932 are most similar to *B. sphaericus *strains 9002, IAB88, IAB872, *Lysinobacillus sphaericus *ISPC-8, H-25 group and 2297. This result is the same as what previously reported by many authors Aquino de Muro and Priest, 1994; Bei *et al.*, 2006; Hu *et al.*, 2008.

Toxicity for mosquito larvae has been associated with the formation of toxic proteins during sporulation and/or vegetative growth. As revealed by SDS-PAGE, the local strains (*Bacillus sphaericus *EMCC 1931 and EMCC 1932) have relatively similar protein profiles (20- to -139 KDa) either during vegetative growth or sporulation with minor exceptions. This finding, to some extent, is in accordance with that obtained early by Baumann *et al. *(1985). They found that solubilization of the preparations at pH 12 with NaOH led to the elimination of all high molecular mass bands but 43- and 63-KDa were not. Other than recently, Smith *et al. *(2005) observed the presence of 110 and 125-KDa SDS-PAGE bands from NaOH extracts of washed *B. sphaericus *spores.

The binary toxin produced by *B. sphaericus *may be synthesized as 110- 125-KDa protein which is converted to a non-toxic 63-KDa moiety and a toxic 43-KDa moiety during the process of sporulation. Additional studies have also shown that the 110- and 125-KDa bands are attributable to the binary toxin by reaction with anti-binary toxin antibodies. Also, a 40-KDa protein was related to 43-KDa toxin Baumann *et al.*, 1985; Broadwell and Baumann, 1986; Shanmugavelu *et al.*, 1998; Smith *et al.*, 2005.

Recently, screening of proteins produced by some toxic isolates of *B. sphaericus *revealed the presence of a ~ 49 kDa protein in spore/crystal preparations. The absence of this protein in other toxic ones to which mosquito resistance has developed, led to the proposal that this might represent a new toxin that could have an important role in the prevention of insect resistance Yuan *et al.*, 2003; Jones *et al.*, 2007. Our results showed that analysis of protein produced by local strains *B. sphaericus *EMCC 1931 and EMCC 1932 revealed the presence ~ 49 kDa protein.

Low toxic strains, synthesize toxic proteins during vegetative growth such as 100-, 35.8- and 31-KDa. In the current study, the presence of 100-kDa corresponding to the Mtx1 toxin was observed on SDS-PAGE only in 18 h old culture of EMCC1931 but not in 120 h. Furthermore, the visualized bands at 30.2- to 38.9- KDa bands in 18 h preparations of all strains may be ascribed to the Mtx2 and Mtx3. In the preparations of 120 h, the bands with molecular masses of 30.9- and 31.5-KDa were detected in all strains except *B. sphaericus *1593.

Prominently, the Mtx1 has a different mode of action from the binary toxin, and which make it an alternative toxin to delay or overcome development of resistance to binary toxin Wei *et al.*, 2006. Lately, Rungrod, *et al. *(2009) demonstrated for the first time that Mtx1 and Mtx2 toxins exhibit synergism against resistant *A. aegypti *mosquito larvae.

From the present results, it is clearly evidenced that media, incubation time and *B. sphaericus *strains play a key role in growth, sporulation, protein synthesis and potency. Prolonging cultivation time up to 5 days actualized the maximum lethal activity and sporulation rate in all media tested. A steady increase in spore counts along the cultivation time was observed. Poly medium proved to be the most auspicious medium for the productivity of *B. sphaericus*.

In literature, the exact cultivation time for obtaining the maximum productivity of *B. sphaericus *strains was conflicting. Some workers found that the maximum level of produced toxins was obtained through 18 - 24 h and prolonging cultivation time had never resulted in consistent increase in spore counts or the larvicide yield. However, others observed a progressive increase in sporulation and toxin production with extending incubation time up to 72 h when *B. sphaericus *cultivated in NYS medium or up to 9 days under semi solid cultivation Myers *et al.*, 1979; Obeta and Okafor, 1983; Bourgouin, 1984; Klein *et al.*, 1989; Foda *et al.*, 2003; Prabakaran *et al.*, 2007; Poopathi and Abdidha, 2007 and 2008.

The presence of larvicidal activity in purified cell wall of *B. sphaericus *1593, 2297 have been observed and attributed to the imperfect separation between cell wall and the crystals could account for this phenomenon. At the completion of sporulation and toxin synthesis, the bacterial cells lyse and liberate the spore and the attached toxic parasporal body Myers and Yousten 1980; Yousten *et al.*, 1989; Klein et al., 2002. This may explain the increasing of toxicity with prolonging cultivation time up to 120 h in the present work.

The comparison between the tested conventional laboratory media indicated that medium composition has a great effect on the growth, sporulation and biocide production by *B. sphaericus*. P medium was found to be the most propitious medium; the proteins released by local strains (*B. sphaericus *EMCC 1931 and EMCC 1932) in P medium followed by NYS medium were more lethal than that produced in the other tested media. White and Lotay (1980) investigate the nutritional requirements of 27 strains of *B. sphaericus*. They found that four strains were grown and sporulated in a simple chemically defined minimal medium, however, the remaining strains needed vitamins or amino acids as well as purine. They also observed that increasing the acetate concentration did not improve growth. Besides, *B. sphaericus *has several important phenotypic properties, including those of being incapable of carbohydrates utilization and having exclusive metabolic pathways for a wide variety of organic compounds and amino acids Russell *et al. *1989, Alexander and Priest, 1990; Han *et al.*, 2007.

Such uneven findings in this study may be, in part, due to the variations in the composition of the tested media in their contents of carbon and nitrogen sources as well as minerals; and to the actual nutritional requirements by a certain strain. By grouping the composition of the five reference media tested in the current study, all media except GGSE medium containing at least two organic nitrogen sources of beef extract, peptone and yeast extract; three of them supplemented with glucose, glycerol or acetate as carbon source; some containing sodium chloride while the others containing Mg^2+^, Mn^2+^, Ca^2+^, Fe^2+^, Zn^2+ ^and potassium phosphates. The calculation of the exact concentrations of carbon and nitrogen (g/l) in each medium used were found to equal, 8.43, 2.4 in P medium; 2.18, 1.22 in NYS medium; 2.03, 0.3 in GGSE medium; 4.13, 2.1 in LB medium and 3.71, 0.98 in AYE medium, respectively. P medium has the highest concentrations of C and N which interprets, in part, the highest yield obtained when all strains were grown in it. The present results gave an idea about the most effective medium that will be used in further study to optimize the biocide production and to develop a low cost effective production medium.

## Competing interests

The authors declare that they have no competing interests.

## Authors' contributions

FMR suggested the research problem and outlined research plan, interpreted the data, prepared the manuscript and have given final approval of the version for publication. All authors, FMR, WDS, MN and HMF, as a research team, carried out the microbiological experiments, bioassay tests and protein estimation, protein profile analysis, DNA extraction matching and submission the sequencing data in the database of gene bank. HMF carried out statistical analysis and figured data. All authors read and approved the final manuscript.
